# Research on the coupled dynamics and prediction of Macao’s tourism-economy-ecosystem from a policy perspective

**DOI:** 10.1371/journal.pone.0321957

**Published:** 2025-05-12

**Authors:** Xieqihua Liu, Sirui Li, Yi Zhu, Ke Song, Hok Kun Wan

**Affiliations:** Faculty of Humanities and Social Sciences, Macao Polytechnic University, Macao, China; Macau University of Science and Technology, MACAO

## Abstract

A harmonious integration of urban tourism, economy, society, and environmental development is crucial for the sustainable development of urban tourism. This study takes the Macao Special Administrative Region as a case, constructing an evaluation index system and a coordination development model for the tourism industry (TI), regional economy (RE), and ecological environment (EE). The study analyzes the coordinated development of these three systems from 2014 to 2023 and uses the GM(1,1) model to forecast the coordination development trend from 2024 to 2026. The results show that during the observation period, the comprehensive evaluation indices of the TI and EE subsystems exhibited significant growth, particularly in the EE system, which had a marked positive impact on the coupling coordination degree. The RE system, however, experienced a short-term decline due to the impact of the pandemic. Despite that, there is still considerable room for improvement in the coupling and coordination capability between Macao’s TI, RE, and EE. In recent years, the relationship between the three has shifted from an initial stage of gradual imbalance to a primary coordination stage. The forecast indicates that in the next three years, the degree of coupling coordination among Macao’s TI, RE, and EE will increase massively, with the systems becoming more synergistic. By 2026, the coordination is expected to progress from the primary to the intermediate stage. This study reveals the dynamic synergistic relationship among the TI, RE, and EE in Macao through the coupled coordination model, verifies the applicability of the coupled coordination theory in the study of sustainable development of regional tourism, and expands its research connotation in the context of coping with emergencies. At the same time, this paper puts forward three suggestions to promote the multifaceted development of the tourism industry, to promote the optimization of the structure of the regional economy and its multifaceted development, and to build an ecologically civilized city. At the same time, the paper puts forward three suggestions, expecting to provide more in-depth theoretical support and practical guidance for the sustainable development of Macau and other tourist cities.

## 1 Introduction

As one of the most dynamic and rapidly growing industries in the global economy, tourism plays a significant role in driving the development of related industries, creating jobs, and increasing tax revenues. At the same time, tourism is a typical environment-dependent industry that places high demands on the quality of the EE [1]. Owing to its unique historical background, Macao has developed a rich tourism resource characterized by the fusion of Eastern and Western cultures and multiple ethnicities. However, due to its limited geographical space, Macao’s TI faces mighty environmental pressures, and the extent of resource utilization is limited, which exacerbates ecological issues [2]. The growing contradictions among tourism economic development, the allocation of tourism resources, and ecological environmental protection are becoming increasingly evident.

The blossoming of Macao’s TI is closely intertwined with its RE and EE, collectively forming a coupled system of TI, RE, and EE [3]. Specifically, the positive development of the TI can significantly enhance the economic interests of tourism in Macao, ensuring ample funding for environmental protection and construction efforts [4]. However, excessive exploitation of tourism resources and the large-scale discharge of tourism pollutants inevitably lead to the deterioration of Macao’s EE [5], which in turn diminishes the tourism experience for visitors, hindering the sustainable boom of the TI. Economic gain further improves tourism infrastructure, creating more internationally attractive tourism products and providing sufficient financial and technological support for ecological protection [6]. However, it is important to note that prompt economic growth often comes at the cost of environmental degradation, leading to increased resource consumption and intensified pollution, creating bottlenecks for tourism development [7–9]. Conversely, improvements in the EE can optimize the structure of Macao’s tourism products, enhancing its image and international reputation as a tourism destination, while environmental degradation and pollution damage Macao’s tourism reputation, negatively affecting tourists’ destination choices and obstructing the boost of the tourism economy [10].

While the RE establishes the lower limit of industry development and the EE sets the upper limit, the TI coordinates resource allocation, and neglecting any of these aspects will hurt Macao’s overall development. Therefore, how to ensure the healthy development of the TI and RE while properly handling the relationship with the ecological environment is of enormous significance in promoting the sustainable development of Macau’s economy. The coupled coordination model of the TI, RE, and EE constructed in this study can make up for the shortcomings of the DPSIR model in inter-system assessment, and it is also closely related to the United Nations Sustainable Development Goals (SDGs), especially Goal 12 (sustainable consumption and production) and Goal 13 (climate action). By looking at how Macau’s TI, RE, and EE are all connected and work together, we can help the city reach its goals for the Sustainable Development Goals and figure out how to balance the benefits for the economy, society, and the environment as the TI grows.

The main objectives of this study are (1) to construct a scientific and reasonable evaluation index system to quantify the development level of Macau’s TI, RE, and EE; (2) to analyze the coupled and coordinated relationship among the three and their dynamic trends; and (3) to predict the coupled and coordinated degree in the next few years to provide a scientific basis and policy recommendations for the sustainable development of Macau.

The potential contributions of this paper include (1) constructing a systematic study on the coupling and coordination of the TI, RE, and EE and making scientific predictions on their future development trends, providing new perspectives and methods for theoretical research in related fields. (2) Using sustainable development theory in tourism cities in even more ways gives us a new way to think about how resilient and long-lasting tourism cities are when they are in trouble.

## 2 Literature review

Currently, the relationships between TI, RE, and the EE within a region, or between two of these elements, are primarily evaluated by using methods such as the coordinated development theory [11], the Environmental Kuznets Curve [12], the CGE model [13], the STIRPAT model [14], the coupling coordination degree model [15], the economic-energy-environment impact model [16], and the gray GM(1,1) model [17]. Wall was one of the first to elaborate on the effect of tourism activities on the EE [18], while Moulin further developed the concept of integrated development between the TI and the ecosystem [19]. Konan conducted empirical tests demonstrating that the advance of the tourism economy can reduce ecological pressure and suggested that there is a dual influence between the tourism economy and the EE system [20,21]. Additionally, Day analyzed the challenges faced by China and America regarding energy and the environment related to regional tourism sustainability and provided targeted recommendations [22].

Domestic research on the TI, RE, and the EE is quite extensive. Most studies concern the coupling and coordination analysis of two systems, such as the TI and RE, TI and EE, and RE and EE. It is widely acknowledged that the impact of tourism activities on the EE is becoming increasingly visible, and the coordinated development between the tourism economy and the EE is salient for regional sustainable development.

In research on the TI and RE, Nikolaos used a panel vector model to conduct an in-depth study on the relationship between economic development and tourism in destinations worldwide. He found that tourism growth driven by economic factors was particularly significant in developing countries and countries with lower levels of tourism specialization [23]. Similar research methods have been widely applied to studies on the relationship between the TI and the RE in various regions. Using panel data from Pacific Island nations, Paresh examined the relationship between real GDP and the TI, noting that a 1% increase in the TI would lead to a 0.72% increase in GDP. In addition, studies by Petrosillo et al. and Lacitignola et al. found that inbound tourism in Eastern China had a prominent effect on regional economic growth, with the growth of inbound tourism being a key driver of the tertiary industry’s growth [24–26]. They proposed strategies for accelerating the TI based on local conditions.

In the research on the relationship between RE and EE, scholars have focused more on the interactive relationship between RE and EE. Many have used coupling coordination models to conduct deep-going studies from various perspectives, such as ecological vulnerability and poverty-stricken areas [27], environmental pollution and economic growth [28], and EE and urbanization [29]. For example, Lai et al.measured the development relationship between the EE and the RE in the TI across 31 provincial cities in China from 2003 to 2017 [3]. Yuan et al. applied the coordinated development model to study the coordinated relationship between the regional environment and RE in the TI of western Hunan, China [30]. They pointed out that the development direction should focus on building a sustainable TI. Furthermore, Zhu et al. used a mixed-effects gravity model to analyze the relationship between inbound tourism and RE in China [31], emphasizing the importance of integrating environmental sustainability into tourism development strategies.

In the TI and EE, scholars have increasingly studied tourism’s ecological efficiency and interaction with the RE. León et al. manipulated the STIRPAT Model to analyze the relationship between the TI and the EE in developed and developing economies, finding that tourism development considerably increases carbon emissions in developed economies [32]. Meanwhile, Dogan et al. confirmed that energy use and tourism development contribute to increased carbon dioxide emissions, while increased trade volume can improve environmental quality [33]. Moreover, Arbulú et al. used panel data from EU countries and found a significant relationship between the TI and environmental degradation [34]. These studies suggest that as RE increases, tourism development tends to have a more significant negative impact on developed economies, highlighting the need for further research and analysis on Macao.

In summary, current research provides theoretical support and methodological tools for studying of the sustainable booming of Macao’s TI. However, the existing literature focuses on the two-way relationship analysis between the RE, EE, and the TI, and it lacks comprehensive studies integrating all three systems. In addition, most studies have focused on the examination of cross-sectional data or short-term development cycles and have seldom dealt with predictive analyses of the coordinated future development of the three systems, making it difficult to comprehensively assess the sustainability of future development. Hence, this paper takes Macao as the study object, uses the entropy method to determine the weight of evaluation indicators, and constructs a coupling coordination degree model for the TI, RE, and EE. Through empirical research, this study analyzes the coupling coordination relationship among the three systems and uses the GM(1,1) gray prediction model to forecast the future coupling coordination trend in Macao. The goal is to provide feasible suggestions for the sustainable development of Macao’s TI.

## 3 Materials and methods

### 3.1 Overview of the study area

Macao Special Administrative Region (SAR) is a part of Chinese territory situated on the southeastern coast of China, lying in the mouth of the Pearl River Delta. It enjoys a subtropical monsoon climate with abundant sunshine and rainfall. The total annual sunshine duration is 1,960.5 hours, the average annual temperature is 26.4°C, and the annual precipitation reaches 2,176.8 mm. Macao’s unique historical background and cultural diversity have endowed it with rich tourism resources. Looking ahead, Macao has positioned itself as a global center for tourism and leisure, setting this as a long-term development goal and striving to soar up the sustainable development of its TI. Since its return to China, Macao’s TI has shown vigorous growth, becoming a key driver of the rapid socio-economic development of the region.

According to data from the Macao Statistical Yearbook and other relevant sources, during the outbreak of the COVID-19 pandemic, Macao’s TI was thriving. Although the pandemic led to a dramatic decline in the number of tourists visiting Macao during the three years of COVID-19, tourism quickly regained vitality with the gradual easing of pandemic control in 2023. Specifically, the number of inbound tourists in 2023 rebounded to 28.213 million, reaching 71.6% of the amount seen in 2019. Meanwhile, the GDP increased to 379.478 billion MOP (Macao Pataca), 85.36% of the GDP in 2019. In addition, the per capita expenditure of tourists increased from 1,665 MOP in 2015–2,515 MOP in 2023. With the full resolution of the pandemic and the high-quality development of the national economy, more tourists are expected to choose Macao as a tourist destination, further promoting the prosperity of Macao’s TI. Tourism has become a vital engine of Macao’s economic development, driving the region’s sustained economic growth.

### 3.2 Measurement methods and principles of development level

The quotient value method is a method of weight allocation based on the relative advantages of the indicator data, which determines the weight of each indicator by calculating its quotient value (i.e., the ratio of the indicator value to the optimal value), thus avoiding the arbitrariness of subjective assignment of weights and being able to reflect the importance of the indicator in the evaluation system more objectively. The entropy method avoids the subjectivity of artificial weights and information overlap between multivariable. It aims to determine the weights of the indicators for the TI, RE, and EEt and calculate the comprehensive scores of these three systems in this study.

In this context, *x*_*ij*_ means the score of the *j-th* factor in the *i-th* year. The specific steps of the entropy method calculation are as follows:

Standardization of Raw Data: The raw data is standardized to resolve the logarithmic issues involved in the calculation of the entropy method. This is achieved by shifting the standardized values to ensure they are within a manageable range for further analysis.


$$\begin{array}{c} {x^{\prime }}_{ij}=\frac{x_{ij}-min(x_{ij})}{min(x_{ij})-min(x_{ij})} \end{array}$$ (1)

(i=1,2,3,⋯n;j=1,2,3,⋯m

To calculate the weight *P*_*ij*_ of the *j-th* indicator in the *i-th* year:


$$\begin{array}{c} P_{ij}=\frac{{x^{\prime }}_{ij}}{\sum_{i=1}^{n}{x^{\prime }}_{ij}}\;(j=1,2,3,\cdot \cdot \cdot m) \end{array}$$ (2)

To calculate the entropy value *e*_*j*_ and the coefficient of variation *g*_*i*_ for the *j-th* indicator


$$e_{j}=-\frac{1}{ln(n)}\sum_{i=1}^{n}p_{ij}log(p_{ij})$$ (3)

($g_{i}=1-e_{j}\;1\leq i\leq m,0\leq e_{j}\leq 1$)

To calculate the *W*_*j*_ of the *j-th* indicator:


$$\begin{array}{c} {\text{W}}_{\text{j}}=\frac{{\text{g}}_{\text{i}}}{\sum_{\text{j = 1}}^{\text{m}}{\text{g}}_{\text{j}}}\;(\text{j}=1,2,3,\cdot \cdot \cdot ,\text{m}) \end{array}$$ (4)

$$\begin{array}{c} {\text{S}}_{\text{i}}=\sum_{\text{j = 1}}^{\text{m}}{\text{W}}_{\text{J}}{\text{P}}_{\text{ij}}\;(\text{i}=1,2,3,\cdot \cdot \cdot ,\text{m}) \end{array}$$ (5)

Finally, the evaluation model for the progress of Macao’s TI, RE, and EE is shown in Equation (5). In this model, *m* represents the number of common factors, *e*_*j*_ is the entropy value, *g*_*i*_ is the standard deviation coefficient, *W*_*j*_ is the weight of each factor indicator, and *S*_*i*_ is the aggregate score. Based on this evaluation model, the comprehensive development scores for Macao’s RE, TI, and EE from 2012 to 2023 can be calculated.

### 3.3 Coupling coordination model

Coupling indicates the phenomenon where two or more systems or forms of motion interact and influence each other. When the components or subsystems of a system work well together and develop in a coordinated manner, it is called a positive coupling. Contrarily, it is called negative coupling when the interaction is poorly coordinated. The coupling degree measures the strength of the interaction and influence between systems or components, while coordination refers to the harmonious and positive cyclical relationship between them [35–37]. Compared with the coordinated development model, the coupled coordination model can not only measure the strength of interactions between systems (coupling degree) but also further assess the level of coordinated development of the system (coordinated development degree) and can intuitively reflect the state of coordination between systems through the hierarchy.

After determining the indicator weights, this study constructs a coupling coordination degree model for the three subsystems: RE, TI, and EE in Macao. The formulas for calculating the coupling association degree and coupling coordination degree of these three subsystems are as follows:


$$\begin{array}{c} C={\left\{\frac{U_{1}\times U_{2}\times U_{3}}{{\left[(U_{1}+U_{2}+U_{3})/3\right]}^{3}}\right\}}^{1/3} \end{array}$$ (6)

In the formula, *C* means the coupling degree, which retrieve values in the range of [0,1]. The greater the value of *C*, the greater the correlation between the three systems. Conversely, a smaller value of *C* indicates weaker interactions between the systems. To better analyze the overall coordination of the systems, this study introduces the coordination development degree model, and its calculation formula is as follows:


$$D=\sqrt{C\cdot T}$$ (7)

$$T=U_{1}\alpha +U_{2}\beta +U_{3}\gamma$$ (8)

In the formula, *D* represents the coordination development degree; *T* is the synthesized evaluation index of the system. The coefficients α、β、γ for the three systems are to be determined, and their sum must equal 1. During the evaluation process, the ecological environment is considered significant to tourism and socio-economic development since the three systems interact in a mutually reinforcing and constraining manner. Referring to existing research [38,39], the coefficients for each system are set to 1/3. Furthermore, based on the classification method by scholar Liao [40], the coupling coordination degree is divided into ten levels (as shown in Table 1).

**Table 1:** Classification of Coupling Coordination Degree.

Range of Coordination Development Degree	Evaluation Level	Range of Coordination Development Degree	Evaluation Level
[0～0.1)	Extremely Imbalanced	[0.5~0.6)	Barely Coordinated
[0.1~0.2)	Severely Imbalanced	[0.6~0.7)	Primary Coordination
[0.2~0.3)	Moderately Imbalanced	[0.7~0.8)	Intermediate Coordination
[0.3~0.4)	Slightly Imbalanced	[0.8~0.9)	Good Coordination
[0.4~0.5)	Borderline Imbalanced	[0.9~1.0]	High-quality Coordination

### 3.4 Grey forecasting model GM(1,1)

The grey forecasting model can utilize a small amount of original data to accumulate the sequence and generate a new sequence. Compared with the ARIMA model, the grey prediction model does not need to assume data smoothness and has low requirements on the distribution pattern, showing stronger robustness; and compared with the machine learning model, it is more suitable for short- and medium-term prediction and fast decision-making scenarios. Therefore, this process helps reduce the original data’s randomness, thereby revealing more distinct characteristic patterns. This study uses the GM(1,1) grey forecasting model to predict the coordinated growth of the TI, RE, and EE in the Macao Special Administrative Region from 2024 to 2027. The specific model is as follows:

Let the original sequence be $x^{0}=\{x^{0}(1),x^{0}(2),\cdots ,x^{0}(n)\}$. After accumulating, the new sequence is $x^{\left(m\right)}=\{x^{\left(m\right)}(1),x^{\left(m\right)}(2),\cdots ,x^{\left(m\right)}(n)\}$, where:


$$\begin{array}{c} \frac{dx^{\left(1\right)}}{dt}+{\alpha x}^{\left(1\right)}=\mu \end{array}$$ (9)

In this context, *a* represents the **development coefficient**, and μ represents the **grey action quantity**.

To further develop the **grey forecasting model GM(1,1)**


$$\begin{array}{c} X^{\left(1\right)}(k+1)=\left[x^{\left(0\right)}(1)-\frac{\mu }{\alpha }\right]e^{-ak}+\frac{\mu }{\alpha } \end{array}$$ (10)

In the grey forecasting model GM(1,1), where *k=(1, 2, ..., n, a* and *μ* are the unknown parameters to be determined, and *k* means the time index.

In order to authenticate the reliability and reasonableness of the prediction results, it is imperative to calculate the posterior error ratio F and the slight error probability *P*, and perform a corresponding test (the accuracy test levels are shown in Table 2). If the posterior error ratio *F* and the slight error probability *P* fall within the acceptable range, the model established for predicting the coupling coordination degree is deemed feasible. Otherwise, it is necessary to analyze the residual sequence and correct to the model formula.

**Table 2:** Accuracy Test Standards for Prediction.

Accuracy Level	Excellent	Qualified	Marginal	Unqualified
F	F≤0.35	0.35<F≤0.5	0.50<F≤0.65	F>0.65
P	P≥0.95	0.80≤P<0.95	0.70≤P<0.80	P<0.70

### 3.5 Data sources and indicator construction

The data for tourism, economy, society, and the environment mainly come from the Macau Statistical Yearbook for 2014–2023 (Table 3). A multi-indicator comprehensive analysis method is employed, with the principles of scientific rigor, practicality, and feasibility. Reviewing and analyzing previous literature [41–42] and drawing upon existing coordination indicator systems, the indicators are classified into three main categories: TI, RE, and EE. Among them, the TI indicators include four indicators as average length of stay of tourists, the average spending of tourists, the total number of tourists and the total spending of tourists, which can reflect the attractiveness of Macao’s tourism and its economic benefits; the regional economic indicators cover four indicators such as the number of travel agencies, annual GDP, per capita GDP and median monthly working income, which can reflect the scale of Macao’s economy and the quality of RE development; and the EE indicators contains four indicators, including average daily sewage treatment, municipal solid waste, population density and motor vehicle density, to measure Macau’s environmental pressure and development potential. This multi-dimensional system of indicators captures the complex interactions and effectively evaluates the coordinated development of Macau’s TI, RE, and EE.

**Table 3:** Coupling Indicator System for TI—RE—EE in Macau.

System Level	Indicator	Unit	Weight	Nature
Tourism Industry System	Average Length of Stay for Visitors	Days	0.0724	Positive
	Average Consumption per Tourist	MOP (Macau Pataca)	0.1597	Positive
	Total Number of Tourists	Ten Thousand Person-times	0.0985	Positive
	Total Tourism Consumption	Million MOP	0.0713	Positive
Economic System	Number of Travel Agencies	Entities	0.1028	Positive
	GDP	Million MOP	0.0841	Positive
	GDP per Capita	MOP	0.0866	Positive
	Median Monthly Working Income	MOP	0.0381	Positive
Ecological Environment System	Daily Wastewater Treatment Volume	Thousand Cubic Meters	0.0528	Positive
	Municipal Solid Waste	Tons	0.0632	Negative
	Population Density	Thousand People/Square Kilometer	0.0631	Negative
	Vehicle Density	Vehicles/Kilometer	0.1074	Negative

## 4 Results

### 4.1 Comprehensive development level analysis of subsystems

**Fig 1:**
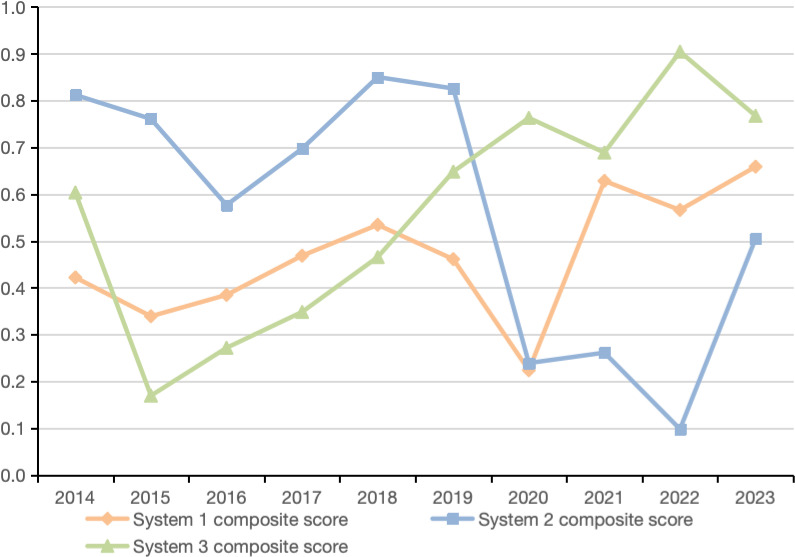
Comprehensive Development Index of Macau’s TI, RE, and EE from 2014 to 2023.

Based on software SPSS 26.0 and the data of various indicators for Macau from 2014 to 2023, this study calculates and analyzes the composite development indices of the TI, RE, and EE subsystems. The specific results are shown in Fig 1.

The TI and EE systems exhibit differing degrees of fluctuating growth. In contrast, the composite development index of the RE system shows a clear downward trend, dropping from a peak value of 0.8503 to a low of 0.0980 before recovering to 0.5049 in 2023. During the study period, all three subsystems—TI, RE, and EE—experienced short-term declines during the fluctuating growth process. Overall, the interconnection among the socio-economic, ecological, and tourism systems has become increasingly tight, and the coordinated development has exhibited a steady upward trend. The detailed analysis is as follows:

Tourism Industry System (System 1): From 2014 to 2023, Macau’s TI experienced a general upward trend with fluctuations. Specifically, from 2014 to 2018, the composite development index of the TI system increased from 0.4220 to 0.5345, reflecting effective adjustments in the structure of the TI and its sustained optimization and development. From 2019 to 2023, the system showed a “V-shaped” fluctuation, with a significant decline in 2020, where the comprehensive development index fell to a low of 0.2237. However, with the gradual relaxation of pandemic restrictions, the Macau TI experienced a strong recovery, and the comprehensive development index notably rebounded, reaching a peak value of 0.6588 in 2023.

Regional Economy System (System 2): From 2014 to 2023, Macau’s RE showed a downward trend with fluctuations. There were two significant periods of decline during the study period, specifically from 2014 to 2016 and from 2019 to 2022, during which the comprehensive development index dropped from a peak value of 0.8503 to a low of 0.0980. The underlying cause of this phenomenon lies in Macau’s high dependence on the TI. The outbreak of the pandemic in 2020 brought Macau a substantial decline in tourists, which sharply impacted the economy, particularly the tourism sector. As the pandemic gradually subsided, the number of tourists to Macau rebounded significantly, effectively facilitating the revival of the TI and, in turn, benefiting the recovery and growth of Macau’s economy. Against this backdrop, the RE system’s composite development index also peeks up to 0.5059.

Ecological Environment System (System 3): From 2014 to 2023, the composite development index of Macau’s EE system showed a continuous upward trend, rising from a low point of 0.1694 in 2015 to a peak of 0.9045 in 2022. Of note is that the development of Macau’s EE system consistently improved, with its composite development index surpassing that of the TI in 2018 and exceeding the RE’s development level in 2020. This indicates that the Macau SAR government has significantly strengthened and improved environmental protection and regulatory mechanisms, effectively promoting the TI’s transition toward a more efficient and low-carbon model. Against this backdrop, the EE quality in Macau has seen marked improvements. In 2023, however, the composite development index of the EE system slightly declined. This was primarily due to the sharp rebound in the number of tourists to Macau, which increased environmental pressures and, in turn, affected the overall score of the EE system.

### 4.2 Temporal analysis of coupling coordination degree

**Fig 2:**
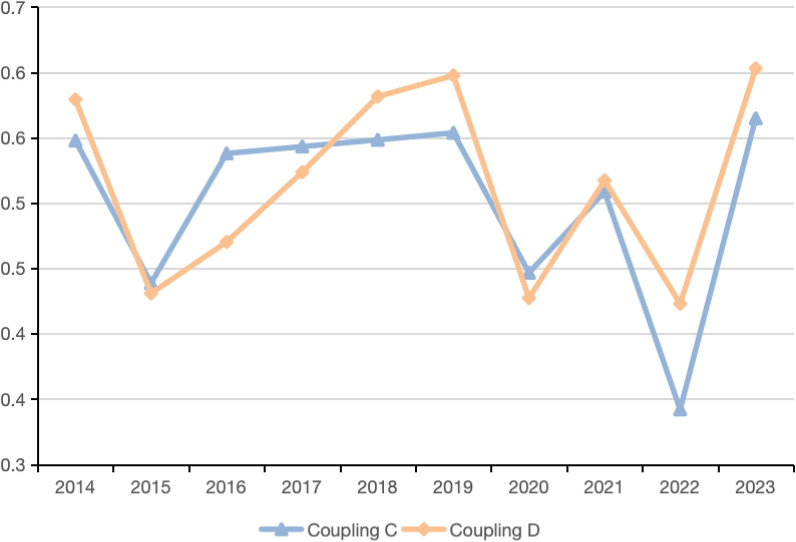
Coupling and Coordination Degree of TI, RE, and EE in Macau, 2014-2023.

According to the comprehensive development indices of each subsystem, the coupling degree (C) and (D) between the TI, RE, and EE in Macau from 2014 to 2023 were calculated. By applying the existing classification criteria, the coupling coordination levels and their specific types for the past decade in Macau can be clearly defined (Fig 2).

From Fig 2, what can be concluded is that the degree of coupling between Macau’s TI, economic development, and EE has generally improved, with the index increasing from 0.5477 to 0.5648. However, during the observation period, the coupling degree exhibited a “W”-shaped fluctuation. Between 2016 and 2019, the coupling degree remained relatively stable, maintaining around 0.5400. However, from 2020 to 2023, a notable upward fluctuation occurred, with the coupling degree rising from the low point of 0.3420 to 0.5648, an increase of 60.55%. This fluctuation trend is mainly attributed to the mutual influence between regional economic and TI factors, which caused the coupling degree to experience significant volatility during this period. Overall, the relationship between Macau’s TI, RE, and EE has strengthened, with their interactions becoming more pronounced and the degree of interaction increasing.

Furthermore, the coupling coordination degree fluctuated in a cyclical wave-like pattern, corresponding to changes in the composite development levels of the TI, RE, and EE systems. The overall coupling coordination degree illustrated a downward and upward trend, ultimately reaching a medium level. During the observation period, the overall coupling coordination degree increased from 0.5792 in 2014 to 0.6030 in 2023. The overall coordination degree hit a low point in 2022, at 0.4220, and then reached its peak in 2023, at 0.6030. This improvement marks the first time the coordination level has reached the “medium” stage. This phenomenon strongly supports the idea that the recovery of Macau’s tourism economy has significantly enhanced the overall development level of the region.

### 4.3 Coupling and coordination type analysis

Due to the changes in the coupling coordination degree, the coordination level of Macau’s TI, RE, and EE has gone through four stages: approaching disorder (2014–2016), barely coordinated (2017–2019), approaching disorder (2020–2022), and primary coordination (2023). Overall, the coordination between Macau’s TI, RE, and EE has gradually improved, with increasing interaction between the systems. The four stages are analyzed as follows:

The first stage is the approaching disorder stage (2014–2016). Macau’s overall coupling and coordination level declined during this period, followed by an increase, reaching a coupling coordination degree of 0.4308 by 2015. In this phase, the growth of Macau’s TI was impacted by tighter transit visa policies and stronger anti-corruption regulations. These measures directly corresponded to the TI, especially the gaming sector, and revealed that the tourism structure needed optimization. Both tourism routes and services were yet to be fully developed. At the same time, the most notable feature of this phase was the quality of the EE, which greatly limited the improvement of the coupling and coordination degree. The environmental challenges and the slow development of tourism caused a diminishment in the coordination between Macau’s RE and the tourism sector during 2015–2016. During this period, Macau’s TI, RE, and EE were mainly approaching disorderly. The Macau SAR government took proactive measures to address these challenges, encouraging gaming enterprises to extend their industrial chains while actively expanding non-gaming elements to diversify Macau’s tourism resources and offerings. This was aimed at attracting a broader range of tourists. Additionally, the government supported the development of the MICE (Meetings, Incentives, Conferences, and Exhibitions) industry to attract more high-end business travelers, stimulating growth across local industries and small to medium-sized enterprises.

The second stage is the **marginally coordinated stage** (2017–2019). During this period, Macau’s overall development index continued to improve, with the composite development indices for the TI and RE showing fluctuating growth, while the composite development index for the EE experienced a significant increase. The coupling coordination degree reached a slight peak of 0.5976 in 2019, approaching the level of primary coordination. In this phase, Macau accelerated its development as a city suitable for tourism and leisure, pushing for the expansion of non-gaming elements by gaming enterprises. It also focused on building a coastal ecological cultural tourism system and further integrated tourism, leisure, and entertainment industries. These efforts continually enriched Macau’s tourism resources. Simultaneously, Macau began cultivating emerging industries and strategically leading economic diversification through innovation. The high quality-rise of the economy had a direct positive impact on environmental improvements. For example, per capita green space increases and daily wastewater treatment capacity led to better environmental quality. These improvements enhanced Macau’s position as an international leisure center, which attracted more tourists and made strides in the overall growth of Macau’s economy.

The third stage is the **precariously uncoordinated stage** (2020–2022). In 2020, the coordination development index dropped significantly from 0.5976 in 2019 to 0.4272, a decline of 39.89%. This drastic change was primarily attributed to the outbreak of the COVID-19 pandemic, which caused the global economy to stall, severely restricted the movement of people, and led to a sharp decrease in the number of tourists visiting Macau. This, in turn, devastated Macau’s TI and overall economy, inducing a dramatic reduction in the degree of coupling coordination. In response to this crisis, the Macau SAR government introduced a series of measures to stimulate domestic demand and rejuvenate the tourism sector. These measures included the launch of electronic consumption vouchers and the “Macau Residents Dining, Accommodation, and Travel” initiative, encouraging residents to explore their communities and experience the city’s diverse tourism offerings. These efforts helped drive local consumption and revive the tourism market. Additionally, Macau actively implemented global green development initiatives, expanding the application of new energy vehicles and accelerating the transition towards cleaner, low-carbon transport in the land transport sector. These initiatives contributed to environmental improvements and supported the burgeoning of a low-carbon TI, paving the way for sustainable growth in the future.

The fourth stage is the **initial coordination stage** (2023). In 2023, Macau’s comprehensive development index showed an improvement. The TI index was 0.6588, a 14.22% increase from 2022. The regional economic index reached 0.5049, significantly higher than the 0.0980 of 2022, recovering to 63.57% in 2019. However, the environmental index dropped by 17.85% compared to 2022. This decline was primarily due to the resurgence in the number of visitors, which, while beneficial for economic recovery, put considerable pressure on Macau’s EE. In response to these challenges, the Macau SAR government has focused on leveraging various central government support measures, continually optimizing cultural tourism products and infrastructure, such as the light rail system. Additionally, the government applied digital technologies to integrate online and offline marketing channels, widely promoting Macau’s tourism resources, increasing the technological content of tourism, and enhancing the overall visitor experience. These efforts support the recovery of the tourism sector and improve Macau’s service quality and tourism innovation, paving the way for sustainable development moving forward.

Based on a comparative analysis of the TI, RE, and EE in Macau between 2014 and 2023, this study categorizes the system states into two main types: economically driven development and ecologically driven development (as shown in Table 4).

**Table 4:** Comprehensive Development Index of Macau’s TI-RE-EE Coordination Development Types (2014–2023).

Year	U1	U2	U3	C	T	D	U1, U2, U3 Comparison Relationship	Coupling Coordination Level	Type Classification
2014	0.4220	0.8121	0.6035	0.5477	0.6126	0.5792	U1＜U3＜U2	Marginal Coordination	Economy-Driven Model
2015	0.3391	0.7609	0.1694	0.4386	0.4231	0.4308	U3＜U1＜U2	On the Verge of Imbalance	Economy-Driven Model
2016	0.3847	0.5768	0.2716	0.5379	0.4110	0.4702	U3＜U1＜U2	On the Verge of Imbalance	Economy-Driven Model
2017	0.4685	0.6973	0.3483	0.5432	0.5047	0.5236	U3＜U1＜U2	Marginal Coordination	Economy-Driven Model
2018	0.5345	0.8503	0.4656	0.5483	0.6168	0.5815	U3＜U1＜U2	Marginal Coordination	Economy-Driven Model
2019	0.4611	0.8259	0.6481	0.5537	0.6451	0.5976	U1＜U3＜U2	Marginal Coordination	Economy-Driven Model
2020	0.2237	0.2391	0.7630	0.4466	0.4086	0.4272	U1＜U2＜U3	On the Verge of Imbalance	Ecology-Driven Model
2021	0.6283	0.2613	0.6889	0.5087	0.5261	0.5173	U2＜U1＜U3	Marginal Coordination	Ecology-Driven Model
2022	0.5662	0.0980	0.9045	0.3420	0.5229	0.4229	U2＜U1＜U3	On the Verge of Imbalance	Ecology-Driven Model
2023	0.6588	0.5049	0.7675	0.5648	0.6437	0.6030	U2＜U1＜U3	Primary Coordination	Ecology-Driven Model

From 2014 to 2019, Macau’s system status was characterized by an economy-driven model, where the comprehensive development indices of the TI and EE lagged significantly behind that of regional RE. This phenomenon highlights that while Macau’s economy experienced rapid growth, it also caused notable negative impacts on the EE. The TI, with its extensive, inefficient, and high-energy-consuming nature, inevitably led to environmental degradation, constraining the EE’s sustainable stride. Furthermore, Macau’s abundant tourism resources have not been fully developed or utilized, and the coordination between the three subsystems—TI, RE, and EE—remained fragile and suboptimal. In the long term, there is still significant growth potential in Macau’s TI and its EE.

Since 2020, the coupling and coordination between Macau’s TI, RE, and EE has undergone a significant shift, moving from an economy-driven model to a more ecology-driven one. During this period, the comprehensive development indices of the TI and RE initially declined before rebounding; however, their overall levels remained much inferior to that of the EE’s comprehensive development index. The primary driver behind this shift was the global impact of the COVID-19 pandemic, which severely restricted population movement and led to a marked cutoff in the number of visitors to Macau, significantly hindering both the tourism sector and the broader economy. To accept these challenges, the Macau SAR government actively embraced national initiatives, continuously advancing industrial transformation, and upgrading while promoting moderate economic diversification. At the same time, the government intensified the introduction of new energy vehicles, effectively reducing transportation energy consumption and enhancing the EE. As the global economy gradually recovers, the sustainable development of Macau’s TI will receive more substantial support and momentum, further contributing to the comprehensive, coordinated, and sustainable progress of Macau’s economy and society.

### 4.4 Coupling and coordination development forecast model

To clarify the future trends in coupling coordination, this study uses the coupling coordination degree sequence of Macau’s TI, RE, and EE from 2008 to 2023 as the original data. Based on the Grey Forecasting Model GM(1,1), the coupling coordination degree for 2024–2026 was predicted. The model calculations yielded the following results for 2024–2026: 0.605, 0.653, and 0.702, respectively. With growing system coordination, the degree of coupling coordination between Macau’s TI, RE, and EE will continue to increase significantly over the next three years. By 2027, the coordination level is expected to shift from primary to intermediate coordination. This reflects the expectation that Macau’s comprehensive development capabilities in tourism, RE, and EE will recover to pre-pandemic levels and suggests that, in the post-pandemic era, it will achieve higher quality development. To achieve this anticipated outcome and promote the evolution of the relationships between the three systems toward higher levels of coordination, the Macau SAR government needs to implement targeted policy measures to overcome the key bottlenecks currently restricting the coordinated development of these three systems. First, the government should address the dominant restrictive factors and accelerate integration into the GBA (Guangdong-Hong Kong-Macau Greater Bay Area) initiative. By collaborating with other cities in the GBA, Macau can achieve deeper integration and enhancement in economic reconfiguration, EE protection, and TI development. Second, it is necessary to focus on environmental protection and sustainable development by establishing an efficient, low-carbon tourism development model. Government, businesses, and residents must collaborate on measures such as green transportation, waste reduction and recycling, and water quality improvement, thereby creating an ecologically sustainable city and minimizing the evil influence of tourism and RE on the environment. Third, efforts should be made to promote economic restructuring and industrial diversification. Macau can foster sustainable economic growth by optimizing industrial policies and encouraging progress in high-tech industries, traditional Chinese medicine, and the health sector. These actions aim to establish a low-carbon, efficient tourism development model, laying a solid foundation for Macau’s economic diversification and long-term sustainability.

## 5 Discussion

This study systematically analyses the coordinated development dynamics of Macau during the period 2014–2023 by constructing a coupled coordination model of the TI-RE-EE. The findings reveal the dynamic changes in the degree of coupled coordination in Macau during this decade and predict the development trend in the next three years. These findings not only answer the research questions but also provide new perspectives for theory and practice in related fields.

### 5.1 Theoretical discussion

This study reveals the dynamic synergistic relationship among Macau’s TI, RE, and EE through a coupled coordination model, which forms an important dialogue with established studies. The results show that the degree of coupled coordination among the three subsystems generally shows an upward trend and gradually transitions from the verge of dysfunction to the stage of primary coordination, which fits with the dynamic evolution law of coupled coordination theory in the established studies [43]. This study further verifies the applicability of the coupled coordination theory in the study of the coordinated development of the TI with the RE and EE [23], which provides empirical evidence for the subsequent related studies.

In contrast to earlier research, this one not only looks at how the TI and the RE work together, but it also includes the EE as a key factor [44], which means that the coupled coordination theory can be used in more areas of tourism. Through system-level comparative analysis, it is found that Macao was in the economic overshooting development stage in 2014–2019, while it shifted to ecological overshooting after 2020, and this shift reveals the reshaping role of major external shocks (e.g., the New Crown Epidemic) on the coupled and coordinated relationship between the TI, RE, and EE [45] and enriches the role of the coupled coordination theory in responding to the the research connotation of the coupled coordination theory in the context of emergencies. In addition, the gray prediction model GM(1,1) was used to scientifically predict the direction of future development. This study fills in the gaps in previous research that didn’t look into long-term trend prediction. The results show that Macau is expected to achieve a higher quality of coordinated development in the post-epidemic era, which is consistent with Zhang’s findings [46] and provides a new perspective and methodology for the dynamic prediction research of coupled coordination theory.

### 5.2 Practical discussion

From a socio-economic perspective, the results of this study reveal the importance of the coordinated development of Macau’s TI, RE, and EE for sustainable socio-economic development. It was found that there is a significant correlation between the fluctuation of Macau’s TI and RE and the continuous improvement of the EE quality during 2014–2023. This indicates that in the process of socio-economic development, the improvement of the environmental environment can not only enhance the quality of life of the residents but also provide strong support for the sustainable development of the tourism industry, which in turn promotes the stable growth of the RE [47]. It is noteworthy that the sharp decrease in the number of tourists during the epidemic significantly reduced the environmental pressure in Macau, especially the problems of traffic congestion and waste emissions were alleviated. This provided a rare opportunity for ecological recovery. The decline in Macau’s Eco-Environmental Index in 2023 as a result of the return of tourists also echoes Sari’s warning of “tourism development-environmental pressure” [48].

Meanwhile, the results of this study provide a scientific basis for the Macao SAR government to formulate strategies for the coordinated development of the TI, RE, and EE. It was found that Macau’s TI and RE are highly dependent on tourism (Lim, 2021), while the improvement of the quality of the EE plays a key role in the improvement of the coupling coordination [49]. Macau has responded to the effects of the Xinguang epidemic better than other tourist cities in China and around the world. They started the e-consumption discount program and the “Macanese Food, Housing, and Tourism” program, and sped up the transition to green transportation. These steps lessened the effects of the epidemic on the TI and the RE, and they helped the tourism market slowly recover. These practical initiatives coincide with the study conducted by Wong [50] and provide valuable lessons for other tourism cities when facing similar crises. In addition, the coupled and coordinated development projections of this study provide confidence and direction for the high-quality development of Macau’s socio-economy. If everything goes as planned, Macau’s coupling coordination degree should reach the intermediate level by 2026. This means that the TI, the RE, and the EE should all grow in a coordinated way after the epidemic. This can be done by continuously promoting policies and improving market mechanisms.

## 6 Conclusions and suggestions

### 6.1 Conclusions

This article establishes an evaluation index system for Macau’s TI, RE, and EE, manipulating the coupling coordination degree model to analyze these three systems’ coupling and coordination development trends. Furthermore, the Grey Forecasting Model GM(1,1) is employed to foresee the coordination status of the three systems from 2024 to 2027. The summaries are as follows:

Firstly, the comprehensive evaluation indices of the TI and EE subsystems have experienced varying degrees of growth, with the EE showing the most significant increase, which has positively affected the coupling coordination degree. However, the regional economic system experienced a short-term decline due to the impact of the global pandemic.

Secondly, The coupling coordination between Macau’s TI, RE, and EE is generally moderate, with significant room for improvement. The coupling coordination degree shows a fluctuating tendency to increase, with considerable volatility. Over the past few years, the coordination capacity of the three systems has gradually shifted from a state of imbalance to primary coordination, indicating that Macau has made progress in balancing economic development and environmental protection. However, further optimization of coordination mechanisms is still required.

Third, During the study period, Macau’s integrated development system alternated between an economy-driven model and an ecology-driven one. From 2014 to 2019, the system followed an economy-driven model, emphasizing the role of economic growth in leading development. From 2020 to 2023, it shifted to an ecology-driven model, highlighting the importance of EEal improvement. The economic system in Macau was growing faster before the pandemic, while improvements in the EE became the focus during the pandemic. Both development trajectories have supported Macau’s branding as an international leisure tourism city, demonstrating the significance of positive interactions between regional RE and the EE for enhancing urban competitiveness.

Fourth, in line with the GM(1,1) grey forecasting model, the degree of coupling coordination between Macau’s TI, RE, and EE will continue increasing substantially over the next three years. The coordination among the systems will gradually strengthen, and by 2027, it is projected to improve from primitive coordination to intermediate coordination. This suggests that the comprehensive development indices of the three systems are likely to recover and surpass pre-pandemic levels, further implying potential progress in Macau’s sustainable development.

### 6.2 Suggestions

Promoting the diversified development of the TI is crucial for its growth. The Macao SAR Government should continue to implement the development strategy of creating a “World Center of Tourism and Leisure” and make full use of Macao’s unique economic advantages to promote the upgrading of the TI. For example, the supporting facilities of scenic spots should be further improved to enhance the overall service capacity and visitors’ experience to strengthen the core competitiveness of Macao’s TI. Based on the pillar role of Macao’s gaming industry, learn from Singapore’s “integrated resort” model, deeply integrate the gaming industry with non-gaming entertainment, vigorously promote the cross-border integration of “Tourism +,” and explore the development of “Tourism + Cuisine. Explore multidimensional tourism innovation models like “Tourism + Gourmet,” “Tourism + Events,” and “Tourism + Conventions and Exhibitions” to help the TI grow, become more competitive, and support the overall steady growth and long-term development of Macao’s economy.

In terms of RE development, Macao should promote the optimization of the RE structure and diversified development. Macao can take advantage of its location as one of the core cities of the Greater Bay Area to actively optimize its economic structure and promote the transformation and upgrading of local industries through deep participation in the division of labour and collaboration of the industrial chain in the Greater Bay Area. On this basis, Macao should also take advantage of its specialty industries to enhance its RE radiation and innovation capacity. At the same time, drawing on Dubai’s “Economic Diversification Strategy”, Macao should actively follow the requirements of the “Macao Special Administrative Region Adequate Economic Diversification Development Plan (2024-2028)” and deepen its tourism and leisure industries and focus on promoting the integration of four key industries, namely, traditional Chinese medicine and big health, modern finance, high-tech, exhibition, trade and culture, and sports. and culture and sports. By continuously optimizing and adjusting the industrial structure, Macao will be able to effectively promote the growth of GDP and the increase of residents’ income, as well as further improve and balance the EE while promoting economic development.

Concerning the EE, the Macao SAR Government should build an ecologically civilized city and reduce the negative impact of the tourism industry and economic development on the EE. The government should increase investment in environmental protection infrastructure, formulate strict environmental protection regulations and standards, and guide enterprises and residents to consciously comply with environmental protection requirements. At the same time, it should promote the green transformation of land transportation, reduce carbon emissions in the transportation sector by promoting the electrification of public transportation and green modes of travel, and strengthen the management of waste recycling and the enhancement of water quality along the coastline to build a clean and healthy EE. The government also needs to strengthen the environmental protection supervision of highly polluting and energy-consuming enterprises, encourage them to improve their technological innovation capabilities and promote their transformation toward intensification and greening. Furthermore, the establishment of a robust environmental protection supervision and assessment mechanism is essential to guaranteeing the successful execution of diverse environmental protection measures and thereby achieving mutual benefits for both economic and ecological aspects.

## 7 Research value, limitations, and future directions

### 7.1 Research value

While researching how Macau’s TI, RE, and EE have all grown together and in sync, it’s clear that, as a small city whose main industry is tourism, Macau shares some traits with other similar tourist destinations:

First, we examine the vulnerability and resilience of a tourism-driven economy. Macao’s economy is highly dependent on tourism, and this single-industry-driven economic model shows obvious vulnerability when the global tourism market is hit by external shocks. Nevertheless, Macao demonstrated strong economic resilience by quickly revitalizing the tourism market after the epidemic. This characteristic is also prevalent in tourism-driven cities such as Singapore. This suggests that tourism-driven economies need to be made more resilient through diversification and policy support while relying on core industries.

Secondly, there is a close connection between the TI and EE. The quality of Macau’s EE has a direct impact on its tourism attractiveness. During the epidemic period, Macao’s ecological pressure was reduced due to a decrease in tourists, and its ecological indicators were significantly improved. The return of tourists after the epidemic put pressure on the EE. This dynamic balance between TI and the EE is also common in tourist cities such as Sanya, Guilin, and Qingdao. This suggests that tourist cities need to focus on ecological and environmental protection while developing the tourism economy to achieve sustainable development.

Third, the significance of industrial upgrading and policy support cannot be overstated. Macao mitigated the crisis during the epidemic through the promotion of moderate and diversified economic development as well as policy support, such as the Electronic Consumption Discount Scheme and the “Macanese Food, Housing, and Tourism” project. Singapore’s development reflects this successful experience of policy guidance and industrial transformation. This suggests that tourist cities need policy support and industrial transformation to cope with external shocks and enhance economic and social sustainability.

### 7.2 Limitations and future research

This study has achieved some results in exploring the coupled and coordinated relationship between the TI, RE, and EE in Macau, but there are some limitations. First, the relatively short period of the data may not adequately reflect long-term trends and potential cyclical changes. The gray prediction model (GM(1,1)), on the other hand, only works with small amounts of data and might not be able to make accurate predictions when dealing with complex, changing systems in provinces and countries. Future research can consider extending the data period to include more years of data to enhance the robustness of the conclusions. Meanwhile, combining multiple prediction models to predict the coupled coordination degree may improve the prediction accuracy. By confronting these limitations and exploring future research directions, we expect to provide more in-depth theoretical support and practical guidance for the sustainable development of Macau and other tourist cities.
